# Sagittal Abdominal Diameter as a Surrogate Marker of Insulin Resistance in an Admixtured Population—Brazilian Metabolic Syndrome Study (BRAMS)

**DOI:** 10.1371/journal.pone.0125365

**Published:** 2015-05-07

**Authors:** Ana Carolina J. Vasques, Roberta S. L. Cassani, Adriana C. e Forti, Brunna S. Vilela, José Carlos Pareja, Marcos Antonio Tambascia, Bruno Geloneze, BRAMS Investigators

**Affiliations:** 1 LIMED—Laboratory of Investigation on Metabolism and Diabetes, Gastrocentro, State University of Campinas, Campinas, São Paulo, Brazil; 2 School of Applied Sciences, State University of Campinas, Limeira, São Paulo, Brazil; 3 Department of Endocrinology, Federal University of Ceará, Fortaleza, Ceará, Brazil; 4 Department of Endocrinology, State University of Campinas, Campinas, São Paulo, Brazil; Virgen Macarena University Hospital, School of Medicine, University of Seville, SPAIN

## Abstract

**Background:**

Sagittal abdominal diameter (SAD) has been proposed as a surrogate marker of insulin resistance (IR). However, the utilization of SAD requires specific validation for each ethnicity. We aimed to investigate the potential use of SAD, compared with classical anthropometrical parameters, as a surrogate marker of IR and to establish the cutoff values of SAD for screening for IR.

**Methods:**

A multicenter population survey on metabolic disorders was conducted. A race-admixtured sample of 824 adult women was assessed. The anthropometric parameters included: BMI, waist circumference (WC), waist-to-hip ratio and SAD. IR was determined by a hyperglycemic clamp and the HOMA-IR index.

**Results:**

After adjustments for age and total body fat mass, SAD (r = 0.23 and r = -0.70) and BMI (r = 0.20 and r = -0.71) were strongly correlated with the IR measured by the HOMA-IR index and the clamp, respectively (p < 0.001). In the ROC analysis, the optimal cutoff for SAD in women was 21.0 cm. The women with an increased SAD presented 3.2 (CI 95%: 2.1-5.0) more likelihood of having IR, assessed by the HOMA-IR index compared with those with normal SAD (p < 0.001); whereas women with elevated BMI and WC were 2.1 (95% CI: 1.4-3.3) and 2.8 (95% CI: 1.7-4.5) more likely to have IR (p < 0.001), respectively. No statistically significant results were found for waist-to-hip ratio.

**Conclusions:**

SAD can be a suitable surrogate marker of IR. Understanding and applying routine and simplified methods is essential because IR is associated with an increased risk of obesity-related diseases even in the presence of normal weight, slight overweight, as well as in obesity. Further prospective analysis will need to verify SAD as a determinant of clinical outcomes, such as type 2 diabetes and cardiovascular events, in the Brazilian population.

## Introduction

The increase in life expectancy in developed and emerging countries and the current global obesity epidemic have resulted in increases in both the incidence and the prevalence of chronic diseases [1]. Insulin resistance (IR) has been recognized as a common underlying component in the genesis of obesity-related diseases, such as type 2 diabetes, hypertension, metabolic syndrome and coronary heart disease [2]. The manifestation of IR is characterized by the diminished physiological ability of the insulin hormone to initiate intracellular signaling in different cell types, which results in a decreased insulin-mediated glucose disposal in insulin-sensitive tissues [3].

The development of laboratory methods and clinically applied surrogate markers to assess IR has been the objective of many studies. The gold-standard method to assess IR is the hyperinsulinemic–euglycemic clamp test, an accurate although cumbersome and expensive method that is available only in advanced research centers. Other methods range from a single fasting blood sample for simple indices, such as the HOMA-IR index (Homeostasis Model Assessment—Insulin resistance), to elaborate modeling approaches based on data obtained during intravenous or oral glucose tolerance tests [3]. Based on the relationships established among the total amount of body adiposity, its distribution and its physiopathological effects in IR, the most common clinically applied surrogate markers of IR are represented by inexpensive and noninvasive classical anthropometric parameters of adiposity, such as BMI, waist circumference (WC), waist-to-hip ratio (WHR) [4], [5] and newly emerging parameters, such as neck circumference [6] and sagittal abdominal diameter (SAD) [4], [5], among others.

The SAD or “abdominal height” was first introduced in 1988, when Kvist et al. [7] demonstrated a good correlation between visceral adipose tissue volume and SAD assessed by computed tomography imaging. In 1996, SAD, anthropometrically measured, was proposed as a surrogate marker of visceral fat mass [8], [9], which has recently become more established [10–12]. Some studies also demonstrated a better performance of SAD compared to waist circumference in the assessment of visceral fat [13], [14] and epicardial fat [15]. Moreover, SAD was considered an independent predictor of sudden death [16] and arterial stiffness [17]. Recently, a well-designed study demonstrated that SAD can predict incident type 2 diabetes [18]. The SAD can also be applied as a screening tool in clinical research for the assessment of cardiovascular risk factors [13], [14], [19–22]. Furthermore, studies conducted with different ethnicities demonstrated close associations between SAD and IR [4], [5], [14], [23], [24]. Currently, one major obstacle for SAD utilization is the lack of widely acceptable reference ranges or cutoff values to assign to the risk categories of SAD.

There is a broad spectrum of IR and different patterns of intra-abdominal fat deposition across ethnicities [25]. The utilization of SAD for the screening of IR requires specific validation for each race. Considering that the Brazilian people are one of the most ethnically admixtured populations in the world, the clinical relevance of IR in the current epidemiological scenario and the promising findings related to SAD, the aims of the present study were 1) to investigate the potential use of SAD compared with other anthropometric parameters (BMI, WC, WHR) as a surrogate marker of IR, determined by the clamp method and by the HOMA-IR index in a large population-based sample of women, and 2) to establish the cutoff value of SAD for screening for IR.

## Materials and Methods

### Subjects

This study was performed as part of the Brazilian Metabolic Syndrome Study (BRAMS), a multicenter population survey on metabolic disorders, which included subjects from different States of Brazil: São Paulo [Campinas (66%) and Itu (12%) cities], Ceará [Fortaleza city (7%)] and Minas Gerais [Três Corações city (5%)]. From 1998 to 2013, a total of 5,668 subjects were included in the study. The sample was selected using an intentional non-probabilistic sampling. The subjects invited to participate were selected from outpatient clinics for type 2 diabetes, metabolic syndrome and obesity or through local advertisements. Healthy volunteers were recruited via local and internet advertising.

The data from 824 subjects met the criteria for a convenience sample for the desired analyses: women, aged from 18 to 65 years, a wide range of adiposity (BMI 18.5 to 49.9 kg/m^2^) and non-diabetic according to ADA criteria [26]. None of the subjects were taking any medications that affected the plasma glucose levels or insulin sensitivity. In the current study, to determine the exclusionary criteria, a combination of interviewer-administered questionnaire and laboratory tests were used. The exclusion criteria were as follows: clinical or laboratory evidence of cardiac, renal, liver or endocrine disease; severe systemic disease (e.g., cancer, heart failure); or AIDS, as well as patients who were body builders or professional or amateur athletes, pregnant or lactating.

This study was approved by the Ethics Committee of the State University of Campinas, Brazil. All participants provided written informed consent before participation.

### Anthropometrical and body composition assessment

All the examiners were trained by one single dietitian to perform all the anthropometric measurements. In addition, all the examiners received an illustrated manual of anthropometry developed exclusively for use in the BRAMS. The subjects underwent a standard detailed anthropometric examination by the trained examiners while wearing light clothing and no shoes. Height was determined using a stadiometer fixed to the wall, with a length of 220 cm and subdivided into 0.1 cm. Weight was measured on an electronic digital scale positioned on a flat surface, with a maximum capacity of 200 kg and a sensitivity of 100 g. The SAD was measured to the nearest 0.1 cm after a normal exhalation while the subjects were in a supine position with their knees slightly bent on a firm examination table. The measurement was taken at the umbilicus level using the Holtain-Kahn Abdominal Caliper (Holtain Ltd, Crymych, United Kingdom), a portable sliding-beam caliper [15], [19]. Waist circumference was measured in a standing position by a flexible and inelastic measuring tape (TBW Ltd, Brazil) at the umbilicus level after a normal exhalation without clothing in the measurement area and taking the necessary care not to compress the tissues. Hip circumference was measured at the most salient point between the waist and the thigh [6]. All measurements were taken in duplicate and averaged. Waist-to-hip ratio and BMI were also calculated. The amount of fat-free mass was determined using a bioimpedance analyzer—model BIA 310 (lean body mass SEE = 1.4 kg, r = 0.97), according to the manufacturer’s protocol (Biodynamics Corporation, Seattle, US).

### Insulin Resistance Assessment

IR was assessed by the HOMA-IR index and the hyperglycemic clamp test.

The HOMA-IR index was calculated as follows: ([fasting plasma glucose] x [fasting plasma insulin])/22.5 [27]. Subjects with HOMA-IR values > 2.71 were considered insulin resistant according to a previous cutoff determined for the Brazilian population [28].

The hyperglycemic clamp test provides a measurement of peripheral IR. A previous study had validated the hyperglycemic clamp test against the euglycemic-hyperinsulinemic clamp test and demonstrated that both techniques yield comparable estimates of IR, with a high coefficient of correlation (r = 0.84; p < 0.0001) [29]. In the present study, the hyperglycemic clamp test was selected as the reference method for validating SAD as a surrogate marker of IR.

A subsample of 50 subjects underwent the hyperglycemic clamp test. All the tests were performed at 8 a.m. and after a 12-h overnight fast. The blood glucose levels were raised to the desired plateau (180 mg/dl) for three hours, following a previous detailed protocol [15]. Glucose levels were measured in all of the blood samples. Insulin resistance was calculated by considering the average glucose infusion rate adjusted for free fat mass (GIR_FMM_) during the last hour of the test. The subjects who were in the first tertile of GIR_FMM_ were considered to be insulin resistant.

### Assays

Blood samples were obtained after a 12-h overnight fast and were stored at -20°C for later evaluation. High-density lipoprotein cholesterol (K 015, Bioclin), triglycerides (K 117, Bioclin), uric acid (K 139, Bioclin) and liver enzymes (K 080, Bioclin) were measured by the automated enzymatic and colorimetric methods. Plasma glucose levels were promptly measured in the fasting state and during the clamp tests using a glucose analyzer (YSI 2700; YSI Life Sciences, Yellow Spring, OH, USA) with a CV of 2%. Plasma insulin levels were analyzed using an automated two-site chemiluminescent immunometric assay (Immulite 1000 System; Siemens Health Diagnostics, USA). The intra-assay and inter-assay CVs were 5.2–6.4% and 5.9–8.0%, respectively, for insulin. Adiponectin levels were measured using an ELISA (Quantikine Human Total Adiponectin Immunoassay; Linco Research), the intra-assay and inter-assay CVs were 2.5–4.7% and 5.8–6.9%, respectively; and the coefficients of variation were below 10%.

### Statistical analysis

Statistical analyses were performed using IBM SPSS-Statistics version 20.0. The values are presented as the mean ± the standard deviation for normally distributed data and as the median and interquartile range for variables with a markedly skewed distribution according to the Kolmogorov-Smirnov test. The Spearman’s correlation coefficient was applied for binary correlations, and partial coefficients of correlation were adjusted by age and total body fat mass. The receiver operation characteristic (ROC) curve was built to determinate the optimal cutoff for SAD. The areas under the ROC curve and the 95% confidence interval were also calculated. The diagnosis of IR was defined as a HOMA-IR index value > 2.71 [28] and for clamp index (GIR_FFM_) values in the first tertile. The optimal cutoffs for SAD were calculated based on the highest sum between the sensitivity and the specificity, which is the threshold with the highest accuracy (minimal false negative and false positive results). The associations between the HOMA-IR index and SAD dichotomized, according to the cutoffs determined in the present study, were tested by logistic regression models. The strength of these associations was assessed by the single odds ratios or after adjustments for age and total body fat mass. Significance was set at p < 0.05.

## Results

The clinical and biochemical characteristics of the 824 adult subjects who were studied are shown in Table 1. It is important to note that the comparison between the total sample and the subsample did not show statistical differences for age, BMI, SAD and HOMA-IR index, ensuring the representativeness of the subsample.

**Table 1:** Clinical and metabolic characteristics of the women studied.

Variables	Total sample	Clamp subsample (n = 50)	p
	(n = 824)		
Age (years)	37 (26–47)	39 (29–47)	0.163
BMI (kg/m^2^)	26.8 (23.6–31.1)	26.1 (22.9–31.8)	0.944
Sagittal abdominal diameter (cm)	20.0 (17.8–23.0)	19.3 (17.0–22.3)	0.108
Waist circumference (cm)	91.7 ± 14.6	92.9 ± 15.2	0.528
Waist-to-hip ratio	0.86 ± 0.09	0.86 ± 0.07	0.734
Fat free mass (kg)	46.7 ± 20.4	46.1 ± 7.6	0.636
Systolic blood pressure (mmHg)	116 (110–122)	111 (100–120)	0.353
Diastolic blood pressure (mmHg)	80 (70–80)	80 (70–83)	0.824
Triglycerides (mg/dl)	94 (69–129)	95 (57–123)	0.345
Total cholesterol	188 ± 41	184 ± 30	0.758
LDL cholesterol	111 (89–133)	109 (86–124)	0.416
HDL cholesterol (mg/dl)	51 (44–61)	53 (46–63)	0.270
Glucose (mg/dl)	80 (75–88)	89 (81–96)	0.001
HOMA-IR	1.71 (1.16–2.69)	1.43 (0.94–2.53)	0.093
Adiponectin (μg/ml)	3.44 (2.35–5.31)	3.32 (2.01–5.09)	0.301
Uric acid (mg/dl)	4.10 (3.50–4.80)	3.80 (3.40–4.70)	0.468
Gamma glutamyl transferase (mg/dl)	19 (14–27)	15 (12–22)	0.007
Aspartate aminotransferase (mg/dl)	18 (15–21)	18 (15–21)	0.782
Alanine aminotransferase (mg/dl)	15 (12–20)	15 (1–20)	0.889
GIR (mg.kg^-1^.min^-1^)	—-	7.31 ± 2.95	—-
GIRFFM (mg.kg_FFM_^-1^.min^-1^)	—-	0.17 ± 0.08	—-

The data are the mean ± SD for variables with normal distribution and the median (interquartile range) for variables without normal distribution, according to the Kolmogorov-Smirnov Z test. The *t* test was applied for variables with normal distribution and the Mann-Whitney test was applied for variables without normal distribution. GIR = glucose infusion rate, GIR_FFM_ = glucose infusion rate adjusted for fat free mass.

Table 2 displays the magnitude of the correlations between the anthropometrical parameters studied and the clinical and biochemical variables related to IR. All anthropometrical parameters showed significant correlations, varying from weak to moderate, with the clinical and biochemical variables (p < 0.01). Interestingly, after adjusting for age and total body fat, the correlations weakened, and SAD remained significant for all of the clinical and biochemical variables assessed, including the HOMA-IR index.

**Table 2:** Correlation coefficients between anthropometrical parameters and clinical and metabolic variables with and without adjustment by age and total body fat mass.

Variables	SAD		BMI		WC		WHR	
	r	r*	r	r*	r	r*	r	r*
BMI^a^	0.88†	———	———	———	0.87†	———	0.53†	———
WC^a^	0.87†	0.58†	0.87†	0.57†	———	———	0.77†	0.79†
WHR^a^	0.62†	0.48†	0.53†	0.30†	0.77†	0.79†	———	———
SAD^a^	———	———	0.88†	0.51†	0.86†	0.58†	0.62†	0.48†
Age^a^	0.37†	———	0.32†	———	0.36†	———	0.46†	———
Systolic blood pressure^a^	0.39†	0.17†	0.42†	0.22†	0.35†	0.15†	0.26†	0.12†
Diastolic blood pressure^a^	0.42†	0.14†	0.43†	0.19†	0.38†	0.14†	0.31†	0.11§
Triglycerides^a^	0.32†	0.22†	0.25†	0.09§	0.28†	0.18†	0.31†	0.23† †
HDL cholesterol^a^	-0.33†	-0.21†	-0.31†	-0.19†	-0.32†	†-0.21	-0.31†	-0.22†
Glucose^a^	0.21†	0.11§	0.22†	0.20†	0.22†	0.16†	0.23†	0.09§
Insulin^a^	0.40†	0.22†	0.39†	0.17†	0.35†	0.11§	0.22†	0.14†
Adiponectin^a^	-0.27†	-0.08§	-0.30†	-0.11§	-0.30†	-0.14†	-0.23†	-0.13†
Uric acid^a^	0.39†	0.14†	0.42†	0.15†	0.35†	0.08	0.26†	0.09
Gamma glutamyltransferase^a^	0.35†	0.20†	0.33†	0.17†	0.31†	0.19†	0.33†	0.29†
Alanine aminotransferase^a^	0.27†	0.12†	0.29†	0.14†	0.26†	0.12§	0.26†	0.21†
Aspartate aminotransferase^a^	0.14†	0.09§	0.15†	0.11§	0.12†	0.08	0.19†	0.15†

GIR_FFM_ = glucose infusion rate adjusted for fat free mass, SAD = sagittal abdominal diameter, WC = waist circumference, WHR = waist-to-hip ratio.

^a^ Total Sample, n = 824.

^b^ Subsample, n = 50.

r = Spearman’s correlation coefficient.

r * = partial correlation coefficient adjusted by age and total body fat mass.

†p < 0.001.

§ p < 0.05.

**Fig 1:**
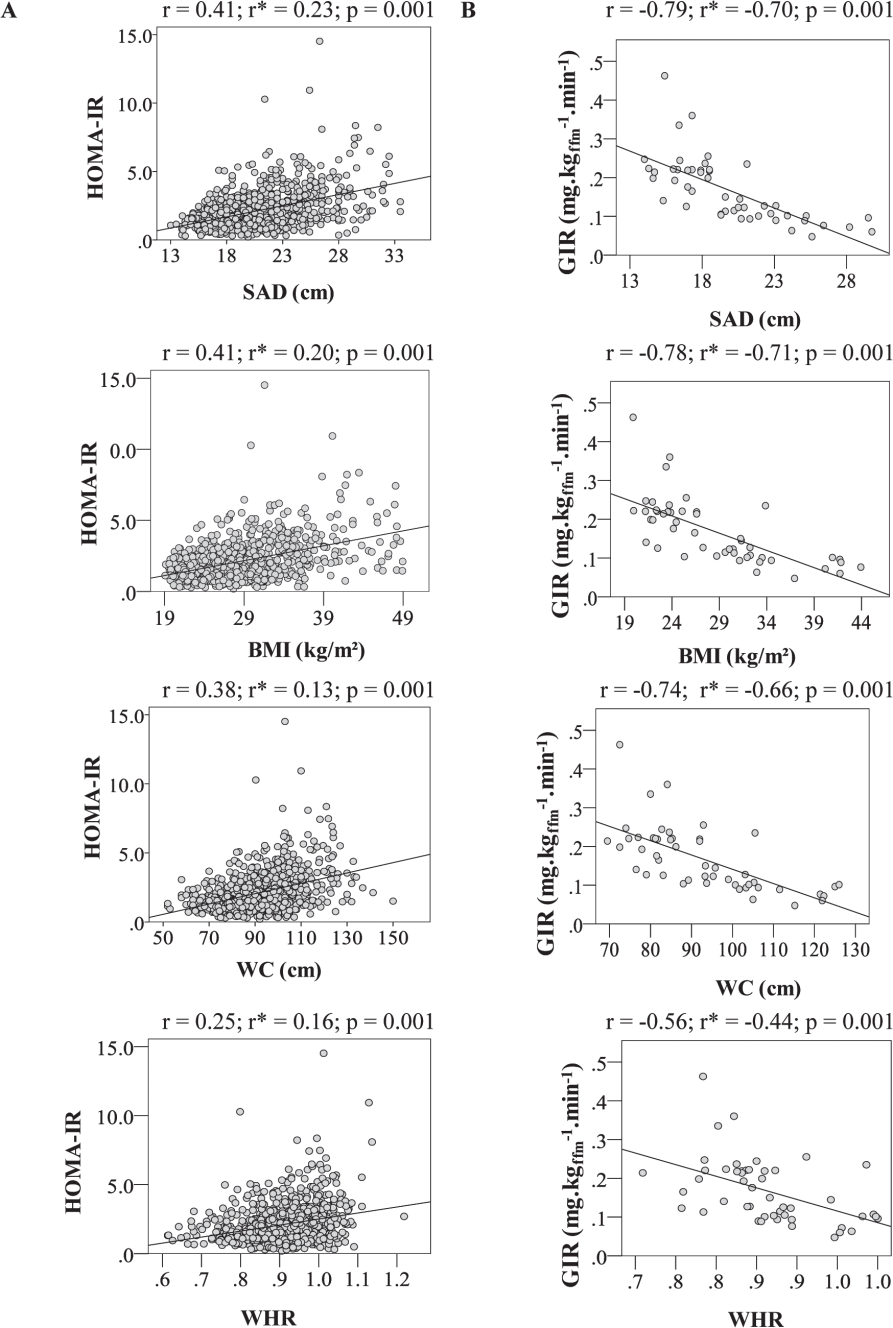
Correlation coefficients non adjusted and adjusted for age and total body fat mass between anthropometric parameters and HOMA-R index (A) and insulin sensitivity index obtained in the hyperglycemic clamp test (B). A: total sample n = 824; B: subsample n = 50. *Adjusted for age and total body fat.

In Fig 1, it is possible to observe that all of the anthropometrical parameters correlated significantly with the IR indices obtained in the fasting state and during the hyperglycemic clamp test, after adjustments for age and total body adiposity (p < 0.001). The strength of correlations ranged from moderate to strong. SAD and BMI presented the stronger correlations with HOMA-IR and GIR_FFM_.

**Fig 2:**
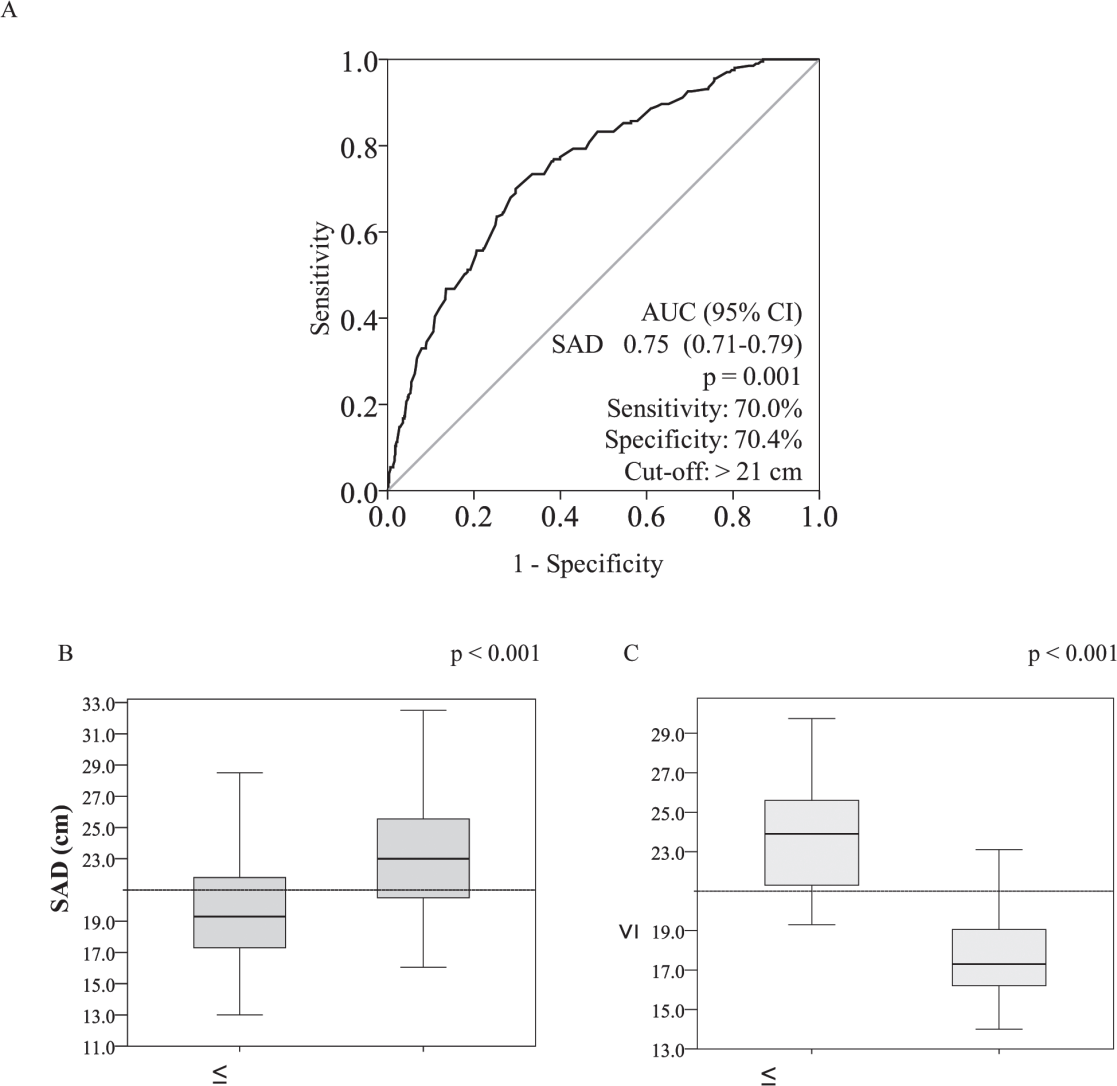
ROC curve for sagittal abdominal diameter for identifying the optimal cutoffs for insulin resistance according to the HOMA-IR index (A) and box plots with the distribution of the sagittal abdominal diameter according to the diagnostic of insulin resistance by the HOMA-IR index (B) and the hyperglycemic clamp test (C). The Mann-Whitney test was applied for SAD.

The Fig 2 illustrates the ROC analyze and the optimal cutoff identified for SAD in women: 21.0 cm.

Finally, to verify the magnitude of the association between SAD and HOMA-IR, both dichotomized, the subjects were classified according to the previously determined SAD cutoffs. For the HOMA-IR index, the results demonstrated that women with an elevated SAD were 5.5 times more likely to have IR compared with women with normal SAD [χ^2^ = 104, odds ratio = 5.5 (95% CI: 3.9–7.8), p < 0.001]. For elevated BMI [odds ratio = 4.5 (95% CI: 3.2–6.3)], WC (odds ratio = 5.1 (95% CI: 3.4–7.6)] and waist-to-hip ratio [odds ratio = 2.1 (95% CI: 1.4–3.3)], women with elevated values were also more likely to have IR compared with women with normal values for these parameters; p < 0.001 for all. Additionally, after adjustment for age and total body fat mass, women with augmented SAD were 3.2 times more likely to have IR compared with women with normal SAD [odds ratio = 3.2 (95% CI: 2.1–5.0); p < 0.001]; whereas women with elevated BMI and WC were 2.1 (95% CI: 1.4–3.3) and 2.8 (95% CI: 1.7–4.5) more likely to have IR compared with women with normal values (p < 0.001), respectively. No statistically significant results were found for waist-to-hip ratio.

## Conclusions

The influence of obesity on the impairment of IR is not only determined by the degree of adiposity but also primarily by the site where the accumulation of fat occurs. Abdominal obesity is strongly associated with IR and metabolic diseases. Although refined and costly laboratory techniques provide an accurate assessment of IR, low-cost and readily available methods are needed for clinical practice and for epidemiologic studies. Among a diversity of anthropometric measurements, SAD has been proposed as a valuable surrogate marker of IR, visceral fat mass and cardiometabolic risk.

The present data are of clinical relevance, as this is the first study that investigated the potential use of SAD for the screening of IR in a large multicenter population-based sample of adults from an admixtured population. We also compared SAD with classical anthropometric measures (BMI, WC, WHR), using the HOMA-IR index and the hyperglycemic clamp test as reference methods. The primary findings of the present study were as follows: 1) all of the anthropometrical parameters correlated significantly with the clinical and biochemical variables related to IR, but SAD and BMI appeared more closely associated with the majority of them, including the IR indices, whereas WHR was more weakly correlated to them; 2) the optimal cutoffs identified for SAD were 21.0 cm for women; and 3) the strength of the association between SAD and IR was reinforced by the significant odds ratios.

The close association between SAD and IR found in the Brazilian ethnic admixtured sample is in line with earlier studies [4], [5], [8], [19], [23], [30]. The magnitude of the correlation between SAD and fasting hyperinsulinemia was initially demonstrated in Caucasians [8]. In the Bogalusa Heart Study’s, in African American and Caucasian participants, SAD was a slightly better predictor of plasma glucose and insulin levels than the other anthropometric measures [31]. A study conducted with non-obese women using the euglycemic-hyperinsulinemic clamp test verified that SAD remained significantly related to IR after adjusting for total body fat [22]. In another clamp study with Caucasian men, SAD presented stronger correlations with IR than BMI, WC and WHR and SAD was the only independent anthropometric predictor of IR [4]. This result was also demonstrated among immigrant women from the Middle East and native Swedes [5]. Two studies with Brazilian adults verified a stronger correlation between the HOMA-IR and SAD compared with WC [12], [14].

In the ROC analysis, SAD demonstrated to be a surrogate marker of IR. In line with our results, Ohrvall et al. [19] also demonstrated a moderate correlation between SAD and fasting insulin in women (r = 0.46; p < 0.05). Petersson et al. [5] demonstrated that SAD explained a greater proportion of the variations in IR even independently of the other anthropometric measures in women. On the other hand, a study conducted with Swedish subjects identified the strongest correlations between SAD and the majority of the cardiovascular risk factors studied for men, whereas in women, the SAD was equal to the WC [22]. In men, it may be explained by the well-established gender dimorphism in regional adipose tissue distribution. In fact, at a given BMI, men present a higher visceral adipose tissue content compared with women [8].

Adiposopathy is characterized by the pathological accumulation of adipose tissue, such as visceral fat, that contributes to the development of an unfavorable metabolic profile, usually accompanied by IR [32]. BMI is widely accepted for the identification of metabolic risk, but when used alone, it does not distinguish adipose tissue from muscle or address body fat distribution. WHR provides information about body fat distribution only partially independent of total body fat, as obese and lean individuals can have exactly the same value of WHR, complicating the interpretation [8]. WC has been claimed to be a strong marker for health risk; however, SAD may carry distinct information beyond that of the other anthropometric parameters. When assessed in the supine position, SAD has been reported as an excellent marker of visceral adipose tissue in many ethnic groups [8], [11], [33], [34], including the Brazilian population [10], [14]. Sampaio et al. [10] demonstrated that visceral adipose tissue as measured through computer tomography was highly correlated with SAD in women (r = 0.80; p < 0.001), followed by WC (r = 0.77; p < 0.001), and WHR (r = 0.72; p < 0.001). Other studies [11], [13], [14] that used computer tomography showed similar results in agreement with Sampaio et al [10].

Previous studies with magnetic resonance imaging demonstrated that in the supine position, visceral abdominal fat tends to elevate the abdominal wall in the sagittal direction. Subcutaneous abdominal fat compresses the abdomen or tends to flow out at the flanks, that is, subcutaneous fat moves to the sides of the waist. For these reasons, variance in the supine SAD might reflect primarily variance in the visceral abdominal fat volume. On the other hand, at the same value of SAD, an increase in WC may reflect an increase in subcutaneous fat storage [9].

SAD, in comparison with WC and WHR, is the only anthropometric measure with high reliability in both lean and overweight subjects [35]. The experience of our group with SAD measurements obtained in the supine position supports a previous report that SAD can be obtained with a high degree of precision [24]. In the present study, SAD was assessed with the legs bent, which improves reliability compared with the measurements of SAD with straight legs [35].

In the present study, the optimal cutoff identified for SAD for screening for IR in women was 21.0 cm. In the Swedish population, Risérus et al. [22] proposed similar cutoff for SAD in women for the identification of an elevated cardiometabolic risk score of 20 cm. A previous study with the Brazilian population indicated that 19.3 cm for women was the optimal threshold value for SAD in the prediction of visceral abdominal fat [10]. The cutoffs identified in screening for IR may be useful in epidemiological research and in clinical practice to identify insulin resistant subjects who would benefit from lifestyle interventions and also to identify those who require a more aggressive intervention.

The limitations of the present study need to be underlined. This is a cross-sectional study, and a further prospective analysis will need to verify SAD as a determinant of clinical outcomes, such as type 2 diabetes and cardiovascular events, in the Brazilian population. Strengths of the study include the large multicenter population-based sample, which covered a wide range of age and adiposity.

In summary, compared with classical anthropometric parameters, SAD, a bedside method, adds another dimension to IR screening and can be considered a suitable surrogate marker of IR. Understanding and applying routine simplified methods is essential, as IR is associated with an increased risk of obesity-related diseases even in the presence of normal weight or slight overweight, as well as in obesity.

## Supporting Information

S1 Dataset Supporting dataset containing clinical, anthropometric and biochemical data of the total sample studied. (XLSX)
