# Correction: Sagittal Abdominal Diameter as a Surrogate Marker of Insulin Resistance in an Admixtured Population—Brazilian Metabolic Syndrome Study (BRAMS)

**DOI:** 10.1371/journal.pone.0134747

**Published:** 2015-07-31

**Authors:** The PLOS ONE Staff

There are errors in Fig 2 that were introduced during the typesetting process. The publisher apologizes for these errors. Please see the complete, correct Fig 2 and its legend here.

**Fig 2:**
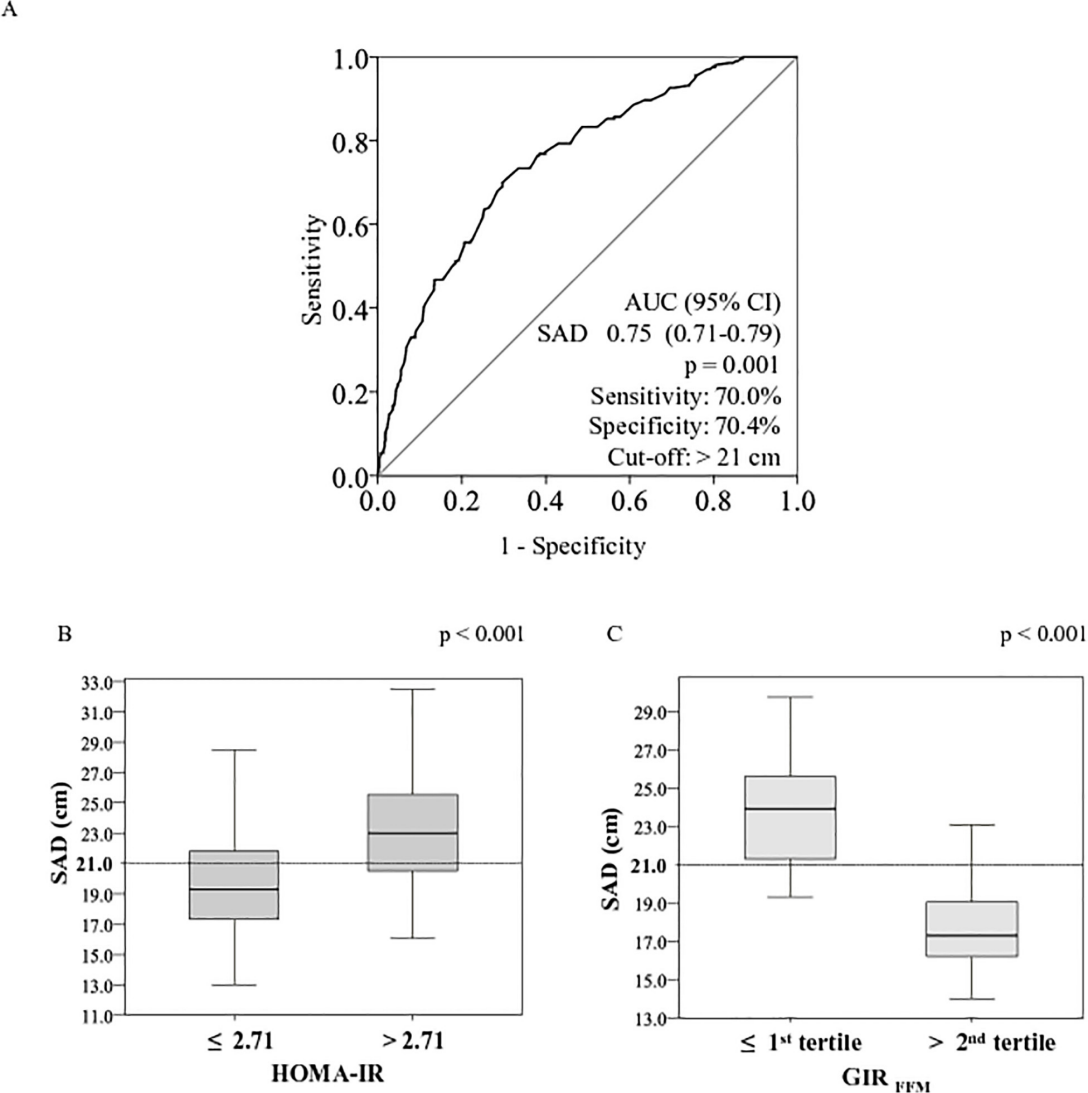
ROC curve for sagittal abdominal diameter for identifying the optimal cutoffs for insulin resistance according to the HOMA-IR index (A) and box plots with the distribution of the sagittal abdominal diameter according to the diagnostic of insulin resistance by the HOMA-IR index (B) and the hyperglycemic clamp test (C).

There are errors in Table 2, “Correlation coefficients between anthropometrical parameters and clinical and metabolic variables with and without adjustment by age and total body fat mass”. Please see the corrected Table 2 here.

**Table 2:** Correlation coefficients between anthropometrical parameters and clinical and metabolic variables with and without adjustment by age and total body fat mass.

Variables	SAD		BMI		WC		WHR	
	r	r*	r	r*	r	r*	r	r*
BMI^a^	0.88†	———	———	———	0.87†	———	0.53†	———
WC^a^	0.87†	0.58†	0.87†	0.57†	———	———	0.77†	0.79†
WHR^a^	0.62†	0.48†	0.53†	0.30†	0.77†	0.79†	———	———
SAD^a^	———	———	0.88†	0.51†	0.86†	0.58†	0.62†	0.48†
Age^a^	0.37†	———	0.32†	———	0.36†	———	0.46†	———
Systolic blood pressure^a^	0.39†	0.17†	0.42†	0.22†	0.35†	0.15†	0.26†	0.12†
Diastolic blood pressure^a^	0.42†	0.14†	0.43†	0.19†	0.38†	0.14†	0.31†	0.11§
Triglycerides^a^	0.32†	0.22†	0.25†	0.09§	0.28†	0.18†	0.31†	0.23†
HDL cholesterol^a^	-0.33†	-0.21†	-0.31†	-0.19†	-0.32†	-0.21†	-0.31†	-0.22†
Glucose^a^	0.21†	0.11§	0.22†	0.20†	0.22†	0.16†	0.23†	0.09§
Insulin^a^	0.40†	0.22†	0.39†	0.17†	0.35†	0.11§	0.22†	0.14†
Adiponectin^a^	-0.27†	-0.08§	-0.30†	-0.11§	-0.30†	-0.14†	-0.23†	-0.13†
Uric acid^a^	0.39†	0.14†	0.42†	0.15†	0.35†	0.08	0.26†	0.09
Gamma glutamyltransferase^a^	0.35†	0.20†	0.33†	0.17†	0.31†	0.19†	0.33†	0.29†
Alanine aminotransferase^a^	0.27†	0.12†	0.29†	0.14†	0.26†	0.12§	0.26†	0.21†
Aspartate aminotransferase^a^	0.14†	0.09§	0.15†	0.11§	0.12†	0.08	0.19†	0.15†

SAD = sagittal abdominal diameter, WC = waist circumference, WHR = waist-to-hip ratio.

^a^ Total Sample, n = 824.

r = Spearman’s correlation coefficient.

r * = partial correlation coefficient adjusted by age and total body fat mass.

†p < 0.001.

§ p < 0.05.
